# Kinetic and Molecular Docking Studies to Determine the Effect of Inhibitors on the Activity and Structure of Fused G6PD::6PGL Protein from *Trichomonas vaginalis*

**DOI:** 10.3390/molecules27041174

**Published:** 2022-02-09

**Authors:** Víctor Martínez-Rosas, Beatriz Hernández-Ochoa, Gabriel Navarrete-Vázquez, Carlos Martínez-Conde, Fernando Gómez-Chávez, Laura Morales-Luna, Abigail González-Valdez, Roberto Arreguin-Espinosa, Sergio Enríquez-Flores, Verónica Pérez de la Cruz, Rodrigo Aguayo-Ortiz, Carlos Wong-Baeza, Isabel Baeza-Ramírez, Saúl Gómez-Manzo

**Affiliations:** 1 Laboratorio de Bioquímica Genética, Instituto Nacional de Pediatría, Secretaría de Salud, Mexico City 04530, Mexico; ing_vicmr@hotmail.com (V.M.-R.); lauraeloisamorales@ciencias.unam.mx (L.M.-L.); 2 Programa de Posgrado en Biomedicina y Biotecnología Molecular, Escuela Nacional de Ciencias Biológicas, Instituto Politécnico Nacional, Mexico City 11340, Mexico; 3 Laboratorio de Inmunoquímica, Hospital Infantil de México Federico Gómez, Secretaría de Salud, Mexico City 06720, Mexico; beatrizhb_16@comunidad.unam.mx; 4 Facultad de Farmacia, Universidad Autónoma del Estado de Morelos, Av. Universidad 1001, Chamilpa, Cuernavaca 62209, Morelos, Mexico; gabriel_navarrete@uaem.mx (G.N.-V.); mcc_ff@uaem.mx (C.M.-C.); 5 Laboratorio de Enfermedades Osteoarticulares e Inmunológicas, Sección de Estudios de Posgrado e Investigación, Escuela Nacional de Medicina y Homeopatía, Instituto Politécnico Nacional, Mexico City 07320, Mexico; fergocha@gmail.com; 6 Posgrado en Ciencias Biológicas, Universidad Nacional Autónoma de México, Mexico City 04510, Mexico; 7 Departamento de Biología Molecular y Biotecnología, Instituto de Investigaciones Biomédicas, Universidad Nacional Autónoma de México, Mexico City 04510, Mexico; abigaila@iibiomedicas.unam.mx; 8 Departamento de Química de Biomacromoléculas, Instituto de Química, Universidad Nacional Autónoma de México, Mexico City 04510, Mexico; arrespin@unam.mx; 9 Laboratorio de Biomoléculas y Salud Infantil, Instituto Nacional de Pediatría, Secretaría de Salud, Mexico City 04530, Mexico; sergioenriquez@ciencias.unam.mx; 10 Neurobiochemistry and Behavior Laboratory, National Institute of Neurology and Neurosurgery “Manuel Velasco Suárez”, Mexico City 14269, Mexico; veped@yahoo.com.mx; 11 Departamento de Farmacia, Facultad de Química, Universidad Nacional Autónoma de México, Mexico City 04510, Mexico; rodaguayo@comunidad.unam.mx; 12 Laboratorio de Biomembranas, Departamento de Bioquímica, Escuela Nacional de Ciencias Biológicas, Instituto Politécnico Nacional, Mexico City 11340, Mexico; charlywong@icloud.com (C.W.-B.); isabelbaeza@yahoo.com (I.B.-R.)

**Keywords:** trichomoniasis, inhibitors, fused G6PD::6PGL, alterations on 3D, docking studies

## Abstract

Trichomoniasis is a sexually transmitted disease with a high incidence worldwide, affecting 270 million people. Despite the existence of a catalog of available drugs to combat this infection, their extensive use promotes the appearance of resistant *Trichomonas vaginalis* (*T. vaginalis*), and some side effects in treated people, which are reasons why it is necessary to find new alternatives to combat this infection. In this study, we investigated the impact of an in-house library comprising 55 compounds on the activity of the fused *T. vaginalis* G6PD::6PGL (TvG6PD::6PGL) protein, a protein mediating the first reaction step of the pentose phosphate pathway (PPP), a crucial pathway involved in the parasite’s energy production. We found four compounds: JMM-3, CNZ-3, CNZ-17, and MCC-7, which inhibited the TvG6PD::6PGL protein by more than 50%. Furthermore, we determined the IC_50_, the inactivation constants, and the type of inhibition. Our results showed that these inhibitors induced catalytic function loss of the TvG6PD::6PGL enzyme by altering its secondary and tertiary structures. Finally, molecular docking was performed for the best inhibitors, JMM-3 and MCC-7. All our findings demonstrate the potential role of these selected hit compounds as TvG6PD::6PGL enzyme selective inhibitors.

## 1. Introduction

Trichomoniasis is a sexually transmitted disease (STD) caused by the protozoan *Trichomonas vaginalis* (*T. vaginalis*). This disease is considered a global health problem, with around 270 million people affected per year, and has an estimated prevalence of 8.1% for women and 1.0% for men [1,2,3,4]. Trichomoniasis treatment is based on use of the nitroimidazole family of drugs, including metronidazole, tinidazole, ornidazole, secnidazole, carbimazole, nimorazole, and satranidazole [5,6,7]. However, there are reports describing drug resistance, and most available drugs have significant side effects that restrict their use [8,9,10]. Based on the above, novel drug design therapies are urgently needed against this parasite.

*T. vaginalis* uses carbohydrates as its primary energy source through its metabolism to glycolysis. In addition, hydrogenosomes are organelles involved in energy metabolism, where the pyruvate is oxidized to produce ATP [10,11]. Another important pathway in the metabolism of *T. vaginalis* is the pentose phosphate pathway (PPP), which is involved in the generation of reduced nicotinamide-adenine-dinucleotide phosphate (NADPH). NADPH plays a crucial role in the viability of these parasites since it participates as an electron donor in biosynthetic processes and in the defense against oxidative damage caused by the host. In addition, the PPP provides nucleotide precursors, such as ribose 5-phosphate, to synthesize nucleic acids and metabolic intermediates, such as fructose-6-phosphate and glyceraldehyde-3-phosphate [12].

Glucose-6-phosphate-dehydrogenase (G6PD) is a housekeeping cytosolic enzyme in all life forms, from prokaryotes to animals. It catalyzes the first rate-limiting step of the oxidative phase in the PPP [13,14]. The *T. vaginalis g6pd* gene is fused with the gene that codes for 6-phosphogluconolactonase (GPGL), giving rise to a fused G6PD::6PGL protein. This same *g6pd*::*6pgl* gene fusion codes the first two enzymes of the PPP and has also been observed in *Plasmodium falciparum* and *Giardia lamblia* parasites [15,16,17]. Differences in fused G6PD::6PGL regarding human G6PD (HsG6PD) make this natural parasite fused protein a potential pharmacological target.

Based on the need to find new targets for drug design against trichomoniasis, the main objective of this work was to characterize the effects of an in-house library of 55 compounds on the functional and structural properties of recombinant fused G6PD::6PGL protein from *T. vaginalis*. Our results indicated that four compounds (JMM-3, CNZ-3, CNZ-17, and MCC-7) efficiently inhibited TvG6PD::6PGL by noncompetitive and uncompetitive inhibition. In addition, these compounds induced alterations in the protein’s secondary and tertiary structures. Finally, we also performed molecular docking to predict the potential binding sites of inhibitors on the TvG6PD::6PGL structure. In general, we suggest that these compounds might be used as a new approach to selectively inhibit the G6PD::6PGL of *T. vaginalis*, without affecting the activity of the homologous human enzyme (HsG6PD).

## 2. Results and Discussion

### 2.1. Purification of the Recombinant Fused TvG6PD::6PGL Enzyme

The recombinant fused TvG6PD::6PGL protein was purified using a Ni Sepharose high-performance affinity column (GE Healthcare, Little Chalfont, Buckinghamshire, UK) and analyzed with 12% SDS-PAGE gels. As seen in Supplementary Material Figure S1, a single band with an apparent molecular weight (MW) of 81 kDa was detected, which belonged to the recombinant TvG6PD::6PGL protein. Then, we removed the 6xHis tag in the N-terminal using the site-specific protease, TEVP, as previously reported [18], and used the resulting protein to perform high-throughput screening assays to identify the compounds that inhibited enzyme activity.

### 2.2. Functional Assays

#### 2.2.1. Selection of Compounds That Inhibit the Catalytic Activity of Fused TvG6PD::6PGL Enzyme

High-throughput screening (HTS) assays have been widely used to identify enzyme inhibitors. For example, Preuss et al. [16] identified five inhibitors of the fused *Plasmodium falciparum* G6PD::6PGL protein.

**Figure 1:**
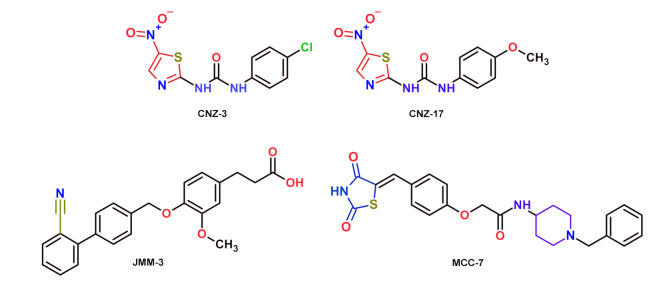
Chemical structures of the selected JMM-3, CNZ-3, CNZ-17, and MCC-7 compounds.

Here, we tested an in-house library of 55 compounds with some structural similarity to the previously reported molecules by Preuss et al. [16]. Table 1 shows ten compounds that inhibited more than 50% of the fused TvG6PD::6PGL enzyme activity at 400 μM. In addition, we tested these synthetic compounds as potential recombinant human G6PD (HsG6PD) protein inhibitors. We observed that synthetic compounds, such as JMM-2, CNZ-3, CNZ-7, and CNZ-8, inhibited the fused TvG6PD::6PGL and the HsG6PD enzymes. For example, JMM-2 inhibited both enzymes by 81%, and CNZ-3 inhibited their catalytic activity by around 90%. Conversely, six of them, JMM-3, CNZ-16, CNZ-17, MCC-7, CMC-1, and TDA-5, inhibited the fused TvG6PD::6PGL enzyme activity in a more significant proportion regarding HsG6PD. The MCC-7 showed an inhibition of 80% of the TvG6PD::6PGL activity, and a low inhibition of 11% on the HsG6PD enzyme. Characteristically, JMM-3 and CNZ-17 showed 56% and 62% inhibition over the fused G6PD::6PGL parasite enzyme, respectively, and they did not inhibit HsG6PD. The selective inhibition over the fused TvG6PD::6PGL enzyme may help improve the rational design of new drugs against this parasite without affecting the activity of HsG6PD. The selected compounds in this study are shown in Figure 1.

#### 2.2.2. Orthogonal Assay

**Figure 2:**
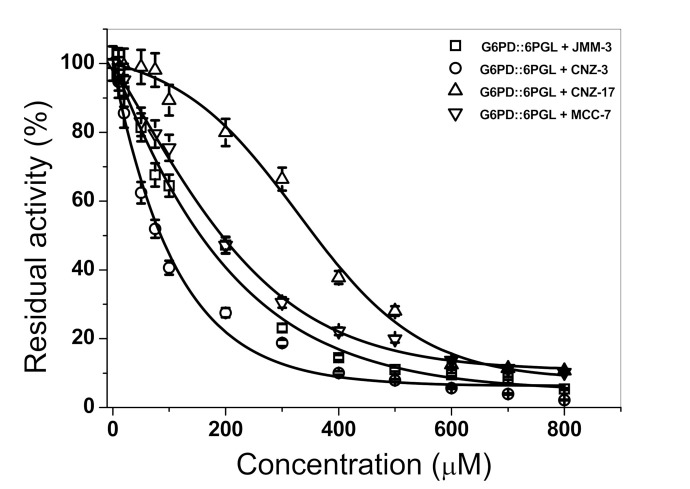
Inactivation of the TvG6PD::6PGL enzyme with CNZ-3, MCC-7, JMM-3, and CNZ-17. The protein was adjusted at 0.2 mg/mL and incubated with increasing concentrations of each compound (0–800 μM) for 2 h at 37 °C. The IC_50_ values were determined by plotting the residual activity of the fused TvG6PD::6PGL enzyme versus compound concentrations. The assays were carried out in triplicate, and the data represent the mean ± standard error.

Subsequently, we determined the IC_50_ values for JMM-3, CNZ-17, and MCC-7 (selective inhibitors of the TvG6PD::6PGL enzyme) and CNZ-3 (an inhibitor of the parasite and human enzymes) (Figure 2). The IC_50_ values determined for CNZ-3, MCC-7, JMM-3, and CNZ-17 were 93.0 µM, 260.1 µM, 155.16 µM, and 356.0 µM, respectively. CNZ-3 showed a higher inhibitory effect at low concentrations, suggesting a higher affinity for the TvG6PD::6PGL enzyme. In contrast, CNZ-17 showed a lower affinity, but a high specificity for the TvG6PD::6PGL protein.

#### 2.2.3. Inactivation of Fused TvG6PD::6PGL Enzyme by Library Compounds

**Figure 3:**
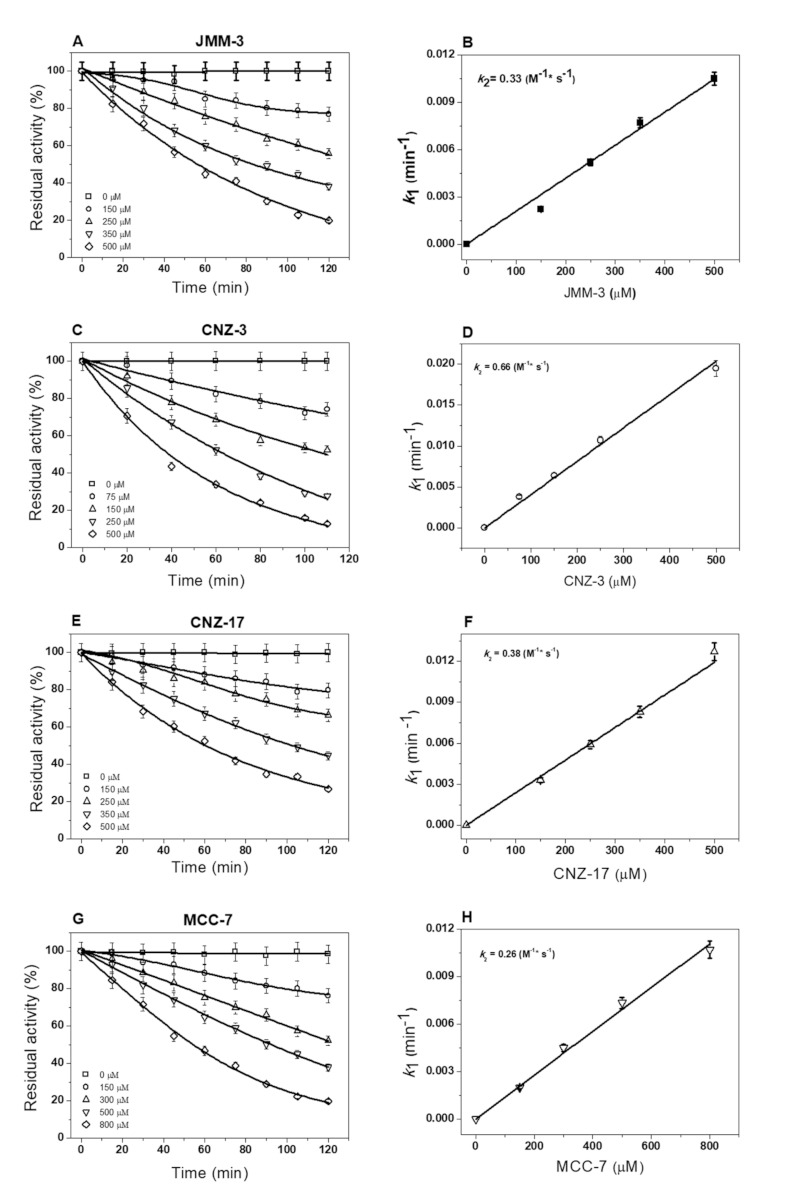
Inactivation of fused G6PD::6PGL enzyme by chemical compounds. TvG6PD::6PGL enzyme was adjusted to 0.2 mg/mL and incubated at 37 °C with different concentrations of (**A**) JMM-3, (**C**) CNZ-3, (**E**) CNZ-17, and (**G**) CCM-7. We fitted initial velocity data using the monoexponential decay equation to determine each compound’s pseudo-first-order inactivation constants (*k_1_*). We obtained second-order rate constant values of inactivation (*k_2_*) of each of (**B**) JMM-3, (**D**) CNZ-3, (**F**) CNZ-17, and (**H**) CCM-7 by fitting the calculated *k_1_* value versus the concentration of the compound. All the experiments were performed in triplicate. The figure shows representative experiments performed in triplicate. The values represent the mean ± standard deviation from three independent experiments, and standard errors were lower than 5%.

To determine the enzyme–inhibitor complex formation rate for each synthetic compound, we first calculated the pseudo-first-order inactivation constants (*k_1_*) by measuring the initial velocities at five fixed concentrations, at intervals between 0 and 120 min. As seen in Figure 3, the four synthetic compounds showed a negative effect on the catalytic activity of the fused parasite protein. We observed single-exponential decays of time-course inactivation for all the compounds examined in this study (Figure 3A,C,E,G). Additionally, as the synthetic compounds’ concentrations increased, the enzyme lost catalytic activity in a shorter incubation time; in contrast, the enzymatic activity of enzymes without compounds remained intact. The *k_1_* values for each compound were calculated and plotted against their concentrations, and a linear behavior was obtained and fitted with the linear equation (Figure 3B,D,F,H). The calculated *k_2_* values for JMM-3, CNZ-3, CNZ-17, and MCC-7 were: 0.33, 0.66, 0.38, and 0.26 M^−1^ s^−1^, respectively. It is important to mention that CNZ-3 formed the enzyme–inhibitor complex faster (*k_2_* value of 0.66 M^−1^ s^−1^), regarding the CNZ-17 compound. This difference in the affinity to form the enzyme–inhibitor complex may be due to the *p*-chlorine moiety present in CNZ-3, unlike CNZ-17, which has a methoxy group in the same position.

#### 2.2.4. Inhibition Type

**Figure 4:**
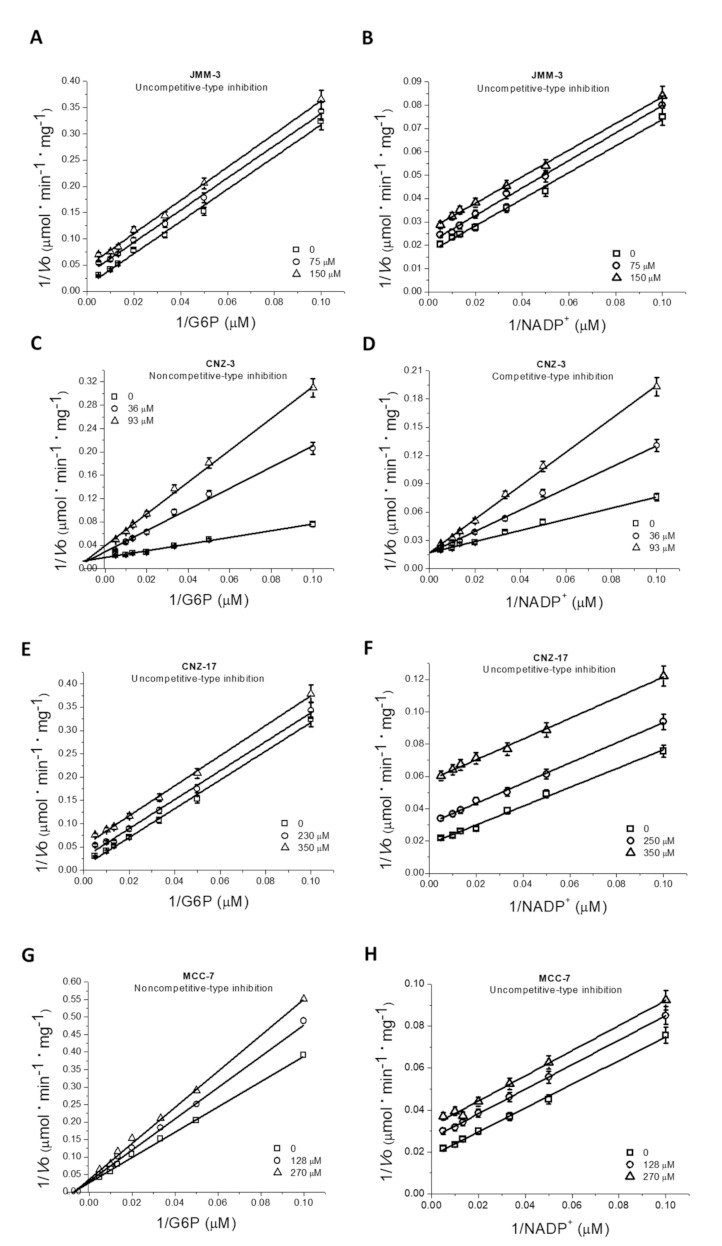
Determination of inhibition type of selected compounds. Lineweaver–Burk plots for the JMM-3, CNZ-3, CNZ-17, and MCC-7 compounds with G6P (**A**,**C**,**E**,**G**) or NADP^+^ (**B**,**D**,**F**,**H**) substrates. The data represent the mean ± of three independent experiments, and standard errors were lower than 5%.

We determined the inhibition type of the four selected compounds over the fused TvG6PD::6PGL enzyme activity (Figure 4). Using double reciprocal plots, we found that JMM-3 showed uncompetitive-type inhibition for both physiological substrates, G6P and NADP^+^ (Figure 4A,B), implying that one or more substrates bind to the enzyme before the inhibitor. For CNZ-3, we observed noncompetitive-type inhibition for the G6P substrate because *V*_max_ decreased in the presence of the inhibitor (Figure 4C); nevertheless, the increased inhibitor concentration did not influence *K*_m_. Meanwhile, for the NADP^+^ substrate, we detected a competitive-type inhibition (Figure 4D). CNZ-17 showed uncompetitive-type inhibition for both substrates, G6P and NADP^+^ (Figure 4E,F). Finally, for MCC-7, we found a non-competitive-type inhibition for G6P (Figure 4G), but an uncompetitive type for NADP^+^ substrate (Figure 4H). It is important to note that only the non-specific TvG6PD::6PGL enzyme inhibitor CNZ-3 showed a competitive-type inhibition for NADP^+^ substrate. In contrast, JMM-3, CNZ-17, and MCC-7, with low or no HsG6PD inhibition, showed noncompetitive and uncompetitive inhibition for both physiological substrates.

### 2.3. Structural Studies

The four selected hit compounds, JMM-3, CNZ-3, CNZ-17, and MCC-7, were used to perform structural assays that allowed us to evaluate whether the loss of catalytic activity was due to alterations on the secondary or tertiary structures (3D), provoked by the binding of the compound to the enzyme.

#### 2.3.1. Circular Dichroism

**Figure 5:**
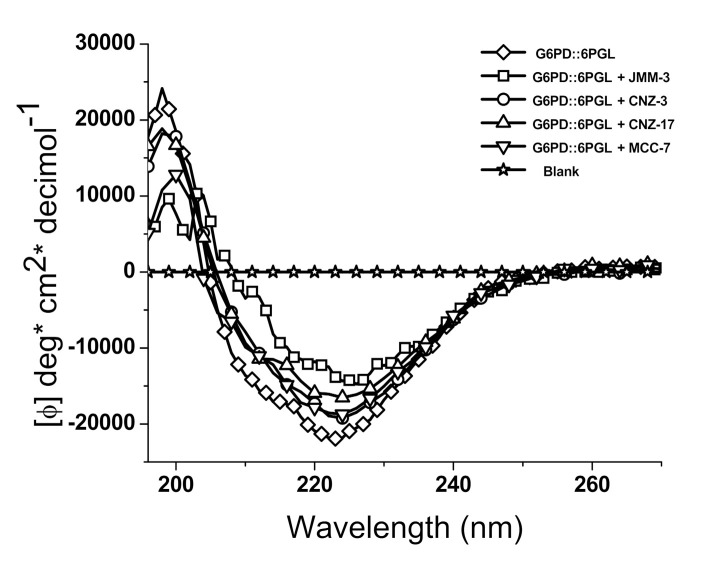
Circular dichroism spectra of the fused TvG6PD::6PGL protein. The four selected hit compounds, JMM-3, CNZ-3, CNZ-17, and MCC-7, were incubated with the protein adjusted at 0.5 mg/mL, and we obtained the spectra in the far UV region from 200 to 260 nm. These experiments are representative of three independent experiments.

We performed circular dichroism (CD) assays to determine if enzymatic activity loss induced by the selected hit compounds was due to secondary structure modifications. As seen in Figure 5, the spectra of the native G6PD::6PGL enzyme have a minimal absorption pattern in the range of 208 to 222 nm, which reflects a protein with α-helical and β-folded conformations. Additionally, we observed that the minimal absorption signals in the presence of the four compounds were lower than in the absence of the compound. According to our results, JMM-3 was the compound that most altered the secondary structure of the parasite enzyme, modifying the minimal absorption signals close to the blank. CNZ-17 was the second compound that exhibited a negative effect on the secondary structure of the protein, followed by CNZ-3 and MCC-17. These results indicate that the four compounds caused changes in molar ellipticity (φ) at 222 nm (α-helix) and 208 nm (β-folded), revealing an increasing amount of random coil, which could explain the loss in catalytic activity. Despite the absence of studies of inhibitors of the fused TvG6PD::6PGL protein, similar findings have been observed over the human G6PD protein, in which compounds that affected catalytic activity also affected its secondary structure [19].

#### 2.3.2. Intrinsic and Extrinsic Fluorescence Assays

**Figure 6:**
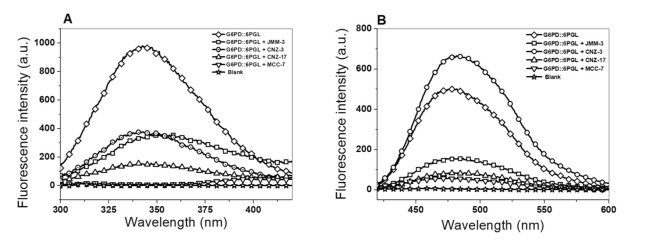
Intrinsic and extrinsic fluorescence assays. Fluorescence emission spectra of the fused TvG6PD::6PGL protein in the absence or presence of the four selected hit inhibitors. (**A**) Intrinsic fluorescence spectra and (**B**) ANS assays of the fused TvG6PD::6PGL protein. TvG6PD::6PGL protein (0.1 mg/mL) was incubated with the IC50 of JMM-3, CNZ-3, CNZ-17, or MCC-7 for 2 h at 37 °C. All assays were carried out in triplicate.

Additionally, we conducted intrinsic and extrinsic fluorescence assays to evaluate inhibitor-induced alterations in protein tertiary structure. As seen in Figure 6A, the four compounds lowered intrinsic fluorescence intensity on the parasite G6PD::6PGL protein. For example, the MCC-7 compound showed the highest negative effect with a maximum fluorescence intensity of 18 arbitrary units (a.u.), representing a 55-fold loss of intrinsic fluorescence intensity, normalized against the protein in the absence of inhibitors (984 a.u.). Additionally, CNZ-17 was the second most effective compound that negatively affected the parasite’s G6PD::6PGL protein intrinsic fluorescence intensity, with 148 au, resulting in a 6-fold intrinsic fluorescence loss, regarding the enzyme without any compound.

Interestingly, an 8 nm redshift was observed in the presence of JMM-3, suggesting the exposure of solvent of previously buried hydrophobic regions. Regarding CNZ-3, we observed a negative 2.5-fold change (382 a.u.) in intrinsic fluorescence intensity. These results indicated that inhibitors caused a rearrangement in the tryptophan residues’ microenvironment, altering the protein’s 3D structure, provoking a loss of catalytic activity. This same finding was reported in a G6PD study, in which a decrease in fluorescence intensity was observed when the human G6PD protein was incubated in the presence of CNZ-3 [19].

Finally, we also evaluated the extrinsic fluorescence signal using the amphiphilic dye 1-anilinonaphthalene-8-sulfonic acid (ANS) to determine alterations in the 3D structure of the fused parasite G6PD::6PGL protein in the presence of the selected hit inhibitors. Characteristically, we found that the CNZ-3 compound induced a 1.3-fold change in the fluorescence intensity compared to the TvG6PD::6PGL protein without any compound (Figure 6B). This increased extrinsic fluorescence intensity suggests conformational changes in the TvG6PD::6PGL enzyme, resulting in more solvent exposure of hydrophobic sites. In contrast, the protein incubated with CNZ-17, MCC-7, and JMM-3 showed maximum fluorescence intensities of 90, 60, and 162 a.u.; these values were below the maximum fluorescence intensity of the G6PD::6PGL enzyme without any inhibitor, about 509 a.u., which represented an extrinsic fluorescence intensity fold change of −5.6, −8.4, and −3.1 (Figure 6B). This decay in extrinsic fluorescence intensity indicated an inhibitor-induced 3D structure protein compaction, diminishing enzyme activity.

In general, the four selected hit compounds caused alterations over the secondary and 3D structures of the fused G6PD::6PGL protein, which explains the loss of catalytic activity. Additionally, we found that the tested inhibitors showed selective uncompetitive and noncompetitive inhibition over the fused TvG6PD::6PGL, making them good candidates for further drug design studies against this amitochondriate parasite.

### 2.4. G6PD::6PGL Model Generation and Selected Hit Inhibitors Molecular Docking

**Figure 7:**
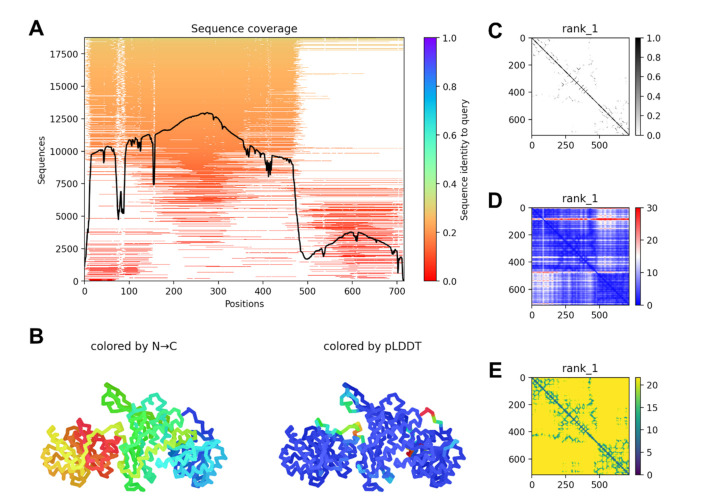
TvG6PD::6PGL model information. (**A**) Multiple sequence alignment coverage. (**B**) Protein model rainbow colored by *N*-terminal to *C*-terminal region (left) and by the predicted local distance difference test (pLDDT) per residue (right). Predicted (**C**) contacts, (**D**) alignment error, and (**E**) distogram of the selected model.

The TvG6PD::6PGL model was constructed using the AlphaFold2 [20] notebook, implemented in the ColabFold Google project [21]. Figure 7A shows the sequence coverage and sequence identity of TvG6PD::6PGL for model generation. Multiple sequence analyses showed that there was a greater identity of the G6PD domain with other sequences than with the 6PGL domain. However, ColabFold was able to generate a reliable model that preserves the structural identity of both domains (Figure 7B). The best ranked model showed several intramolecular contacts and close distances, some of them at the interface formed between the domains, with only a few irregularities in the alignment of the N-terminal region and in the loop that joins both enzyme domains (Figure 7C,D).

For docking experiments, we selected the most effective TvG6PD::6PGL protein inhibitors, JMM-3 and MCC-7. Therefore, we performed molecular blind docking analyses using the entire surface topology of the TvG6PD::6PGL model protein in its monomeric form.

The results obtained by the molecular docking assay with SwissDock for JMM-3 and MCC-7 are shown in Supplementary Table S1 and Supplementary Figure S2. The predicted binding sites were clustered in 31 and 37 clusters for JMM-3 and MCC-7, respectively, with populations of 5–8 members. The cluster rank was predicted by the full fitness energy of the members. The best full fitness corresponded to the first member of each cluster.

Two principal interaction zones were observed; zone 1 was near the binding site of NADP^+^ (Figure 7A). These results confirmed the experimental inhibition assays, which determined both compounds as NADP^+^ uncompetitive inhibitors. In addition, we identified seven amino acids in the protein’s binding pocket, interacting with both inhibitors, Pro135, His139, Pro165, Phe166, Gly167, Thr172, and Asp178 (TvG6PD::6PGL numbering). Interestingly, Pro165, Phe166, and Gly167 are part of the conserved EKPxG peptide in G6PD’s enzymes; this peptide seems crucial for the substrate’s correct approach coenzyme during the enzymatic reaction.

**Figure 8:**
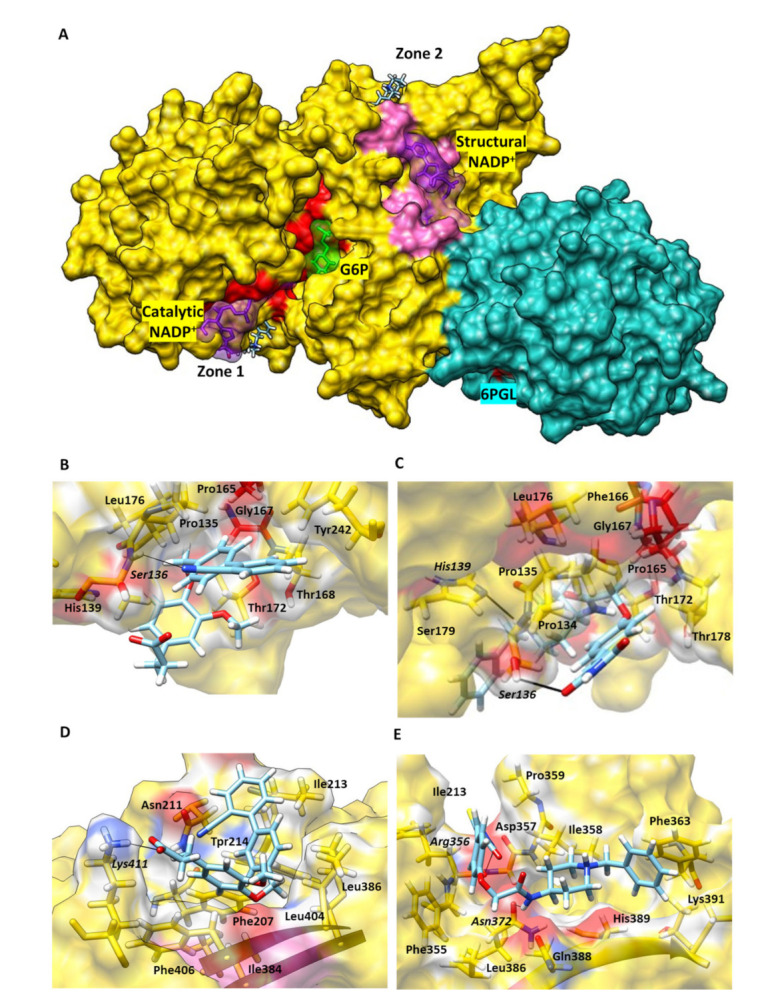
Molecular docking of the selected hit compounds on the TvG6PD::6PGL model. (**A**) General view of the zone 1 and zone 2 binding cavities of the G6PD (gold) and 6PGL (light sea green) on the G6PD::6PGL protein. G6P, NADP^+^, and 6PGL molecules are shown in green, purple, and salmon. (**B**) Zoom on the binding interactions of JMM-3 and (**C**) MCC-7 in zone 1. (**D**) Zoomed in view of the interactions of JMM-3 and (**E**) MCC-7 in zone 2. H-bonds are represented as black lines, and the amino acid residues in bold.

Regarding JMM-3 in zone 1, it interacted with Ser136 via an H-bond, its cyan group, and ten nonpolar contacts (Figure 8B); the most stable protein–ligand complex showed ΔG = −7.08 kcal/mol. MCC-7 formed two *H*-bonds, created between the His139 and the nitrogen of piperidine ring group of this ligand. The second H-bond was formed with Ser136 and thiazolidine’s ring nitrogen. Additionally, we found twelve nonpolar contacts (ΔG = −8.04 kcal/mol) (Figure 8C).

We found a second zone far from the enzyme’s catalytic site for both compounds, called zone 2. Four amino acids in common, Ile213, Trp214, Ile384, and Phe406, interacted with both compounds in this binding pocket. This zone appears located behind the structural NADP^+^ binding site (Figure 8A). The docking suggested that the most stable protein-JMM-3 complex showed a ΔG = −8.65 kcal/mol, forming one H-bond between the Lys411 and the JMM-3 carboxylic acid group (Figure 8D). While MCC-7 formed two *H*-bonds between Asn372 and the nitrogen of acetamide group of MCC-7, and the second *H*-bond was formed with Arg356 and thiazolidine ring (Figure 7E), the most stable conformer showed ΔG = −7.38 kcal/mol (Figure 8E). These results revealed that JMM-3 and MCC-7 probably do not affect the binding of substrates because they are not competitive inhibitors, but they probably affect the correct positioning of the NADP^+^.

## 3. Materials and Methods

### 3.1. Expression and Purification of the Recombinant Fused G6PD:6PGLPprotein

Recombinant *T. vaginalis* G6PD: 6PGL was used to perform the functional and structural assays. *E. coli* BL21(DE3)Δzwf::kanr expression cells containing the expression vector pET3a-HisTEVP cloned with the *g6pd*::*6pgl* gene from *T. vaginalis* were used to overexpress the fused G6PD::6PGL (accession TVAV_414060 obtained from TrichDB database) according to Morales-Luna et al. [18].

The G6PD::6PGL protein was purified from the resuspended cells in lysis buffer (50 mM K_2_HPO_4_; 150 mM NaCl; 2 mM DTT; 0.5 mM phenylmethylsulfonyl fluoride (PMSF) dissolved in dimethylsulfoxide (DMSO), and 1.4 mM β-mercaptoethanol) at pH 7.35. Subsequently, the cells were lysed by sonication, and the crude extract was obtained by centrifugation at 13,000 × *g* for 40 min. Next, the crude extract was loaded onto the Ni-Sepharose affinity column previously equilibrated with equilibrium buffer (EB), 50 mM K_2_HPO_4_, 150 mM NaCl, and 2 mM DTT, at pH 7.35. Subsequently, the column was washed with ten bed-volumes with EB added to 25 mM imidazole. Then, the protein was eluted with the same EB plus 250 mM imidazole. Finally, the fractions with G6PD activity were concentrated and consecutively diluted five-fold using a Centricon-30 kDa centrifugal filter unit (Millipore, Burlington, MA, USA) to remove the imidazole. The protein purity was confirmed with 12% SDS-PAGE gels, stained with colloidal Coomassie blue (R-250) (Sigma Aldrich, St. Louis, MO, USA).

### 3.2. Functional Assays

#### 3.2.1. Selection of Compounds That Inhibit the Catalytic Activity of Fused TvG6PD::6PGL Enzyme

We tested an in-house library of 55 chemical compounds synthesized in the Medicinal Chemistry Laboratory from Facultad de Farmacia, Universidad Autónoma del Estado de Morelos [22,23]. In-house libraries are advantageous in the discovery of novel bioactive small molecules. Purified chemical library members were dissolved in DMSO and incubated at a final concentration of 400 µM, with or without 0.2 mg/mL of the purified TvG6PD::6PGL enzyme, for 2 h at 37 °C. Subsequently, the residual activity was measured using a standard reaction mixture (1 mM G6P, 0.2 mM NADP^+^, 0.1 mM Tris-HCl, and one mM MgCl_2_, pH 8.0). The enzymatic reaction was carried out with 1 µg of the *T. vaginalis* fused protein. The results are shown as the residual activity percentage, in which 100% represents the enzyme’s activity in DMSO with no chemical compound. Finally, we selected compounds that showed more than 50% inhibition.

#### 3.2.2. Orthogonal Assays

Compounds that showed more than 50% inhibition in the recombinant G6PD::6PGL enzyme were used to perform inactivation assays. The compound concentration at which the enzyme loses 50% of its initial activity (IC_50_) was determined according to Hernández-Ochoa et al. [24]. The G6PD::6PGL protein was adjusted to a 0.2 mg/mL concentration and incubated for 2 h at 37 °C, with increasing concentrations from 0 to 800 µM of the selected synthetic hit compounds. Then, the residual activity was measured, as mentioned above.

#### 3.2.3. Second-Order Rate Constant (*k_2_*) of Selected Hit Compounds Showing More Than 50% Inhibition

Second-order rate constants of inactivation (*k_2_*) were used to determine the inactivation rate of a chemical compound and the formation of the enzyme–inhibitor complex. Therefore, pseudo-first-order rate constants (*k_1_*) were determined for the recombinant G6PD::6PGL, adjusted at 0.2 mg/mL, and incubated with four different fixed concentrations of each compound at 37 °C. At the indicated times, a sample was withdrawn, and the residual activity was measured. First, we calculated the pseudo-first-order rate constants (*k_1_*) by fitting the residual activities data using a monoexponential decay equation: $A_{R}=A_{0}\;e^{-kt}$, where *A_R_* is the residual activity at time t, *A_0_* is the activity at the initial time, and *k* is the pseudo-first-order inactivation constant (min^−1^). Then, *k_1_* values for each compound were plotted against the employed concentration of each compound. The second-order rate constants of inactivation, *k_2_* (M^−1^ s^−1^), were obtained from the slopes of the linear plots (*k_1_*) versus the concentration [19,25,26].

#### 3.2.4. Determination of Inhibition Type

To determine the inhibition mechanism of the four selected hit compounds over the *T. vaginalis*-fused G6PD::6PGL protein, we monitored the initial velocities in the presence of two fixed concentrations of each of the inhibitory compounds. The initial velocities were determined at different substrate concentrations (from 0 to 140 µM). In comparison, the second substrate (G6P or NADP^+^) was maintained at a saturated concentration (~5-fold of the *K*_m_ G6P value = 540 μM, and ~5-fold of the *K*_m_ NADP^+^ value = 600 μM). Subsequently, the initial velocities for each physiological substrate (G6P and NADP^+^) were analyzed using the double reciprocal plot method.

### 3.3. Structural Studies

#### 3.3.1. Circular Dichroism Experiments

The secondary structure analysis of the recombinant G6PD::6PGL protein, incubated with the four selected hit compounds, was performed using circular dichroism (CD) in a spectropolarimeter (Jasco J-810^®^, Inc., Tokyo, MD, USA). First, the protein was adjusted at 0.2 mg/mL in 50 mM phosphate buffer, pH 7.35, and incubated for 2 h at 37 °C in the presence of the compounds at IC_50_ concentration. Then, the determination of the secondary structure was carried out in the far UV region (190–250 nm) at intervals of 1 nm in a rectangular quartz cuvette with a path length of 0.1 cm. Spectra of the 50 mM phosphate buffer (pH 7.35) containing each compound were used as blanks and subtracted from all the obtained spectra containing the fused parasite enzyme.

#### 3.3.2. Intrinsic and Extrinsic Fluorescence Assays

To determine the effect of the compounds on the tertiary structure (3D) of the recombinant G6PD::6PGL protein, intrinsic and extrinsic fluorescence assays were performed. Both assays were performed on a Perkin Elmer LS-55 fluorescence spectrometer (Perkin Elmer, Wellesley, MA, USA) [26,27]. In intrinsic and extrinsic fluorescence assays, the protein was adjusted to a protein concentration of 0.1 mg/mL and incubated for 2 h at 37 °C with the IC_50_ of the four selected hit compounds. For the intrinsic fluorescence assay, the samples were excited at 295 nm, with excitation and emission slits of 4.0 nm and 5.0 nm, respectively, and the emission spectra were obtained from 300 to 500 nm. For the extrinsic fluorescence assay, 8-anilinonaphthalene-1-sulfonic acid (ANS) was used, and the samples were excited at 395 nm using slits of excitation and emission of 4.0 and 5.0 nm, respectively. Then, the emission spectra were recorded from 400 to 600 nm. Results are shown as the average of five scans minus the spectra blank (buffer of reaction plus ANS).

### 3.4. Blind Molecular Docking

We performed blind docking using the SwissDock Server (http://www.swissdock.ch/docking, accessed on 10 November 2021) to identify all possible interactions of compounds on the fused G6PD::6PGL protein [28]. Here, we used the G6PD::6PGL model to add the hydrogen and the atomic coordinates, the protein was submitted to the MolProbity server (http://molprobity.biochem.duke.edu/, accessed on 12 October 2021) [29], and system energy minimization was also performed. For the ligands, the 3D structures of the compounds were prepared in ACD/ChemSketch software [30]. Then, the ligand structures were energy-minimized by the Avogadro program (http://avogadro.cc/, accessed on 28 October 2021) [31], and a protonated state was considered if the compounds had an ionic group. Finally, the docking performed in the SwissDock server generated all possible binding modes for each compound, and the most favorable binding modes at a given pocket were clustered in each experiment, a total of 256 poses per ligand were obtained. The predictions file provided cluster rank/element full fitness and estimated binding free energy, ΔG. The affinity energies, the three-dimensional configuration, the formation of hydrogen bonds, the specific atoms involved, and the distance between them were analyzed to select the most stable binding for each compound. In such a way, we selected the pocket with the highest percentage of conformers, with the lowest free energy (ΔG) and with the highest number of hydrogen bonds. The docking results were loaded into and analyzed by Chimera software 1.14.2 [32].

## 4. Conclusions

Trichomoniasis is a sexually transmitted disease that has persisted for many years, so the search for new drugs to combat infections caused by *T. vaginalis* is of great importance. This work studied four selected hit compounds, JMM-3, CNZ-3, CNZ-17, and MCC-7, which showed a remarkable inhibition role over the fused *T. vaginalis* G6PD:6PGL enzyme. These inhibitors induced a change in the protein’s secondary and three-dimensional structure. Particularly, JMM-3 and MCC-7 were uncompetitive inhibitors for NADP^+^ and G6P, and docking revealed that JMM-3 and MCC-7 were not found at the binding site of either G6P or NADP^+^, predicting that the inhibitors bind close to the binding site of NADP^+^, which highlights their role as potential therapeutic drugs since they do not compete to bind to the protein’s active site. In terms of the dose-effect, this allows the use of low compound doses to achieve the desired effect. We suggest that JMM-3 and MCC-7, as TvG6PD::6PGL in vitro inhibitors, are potential candidates for future studies on *T. vaginalis* trophozoites to determine if they negatively alter parasite metabolism.

## Figures and Tables

**Table 1:** Compounds showing more than 50% inhibition over the fused TvG6PD::6PGL enzyme.

Compounds (400 μM)	G6PD::6PGL Inhibition (%)	HsG6PD Inhibition (%)
JMM-2	79	81
JMM-3	56	3
CNZ-3	93	92
CNZ-7	52	68
CNZ-8	51	43
CNZ-16	61	31
CNZ-17	62	0
CMC-1	63	34
TDA-5	54	17
MCC-7	80	11

## Data Availability

Not applicable.

## Supplementary Materials

Figure S1: Purification of the recombinant TvG6PD::6PGL enzyme. Figure S2. Molecular docking of the TvG6PD::6PGL model with compounds obtained with the SwissDock server Table 1. Data obtained by molecular docking assays with the SwissDock server for the most stable clusters.

## Sample Availability

Samples of the compounds are not available from the authors.
