# Supplementary material for: Kinetic and Molecular Docking Studies to Determine the Effect of Inhibitors on the Activity and Structure of Fused G6PD::6PGL Protein from Trichomonas vaginalis
Source: Molecules. 2022 Feb 9;27(4):1174. doi: 10.3390/molecules27041174 (PMC8880039; doi:10.3390/molecules27041174)
Supplement: Supplementary file 1 [file molecules-27-01174-s001.zip › molecules-1556021-supplementary.pdf]

Kinetic, molecular docking, and dynamics studies to determine the effect of inhibitors on the activity and structure of fused G6PD::6PGL protein from *Trichomonas vaginalis*

Víctor Martínez-Rosas [1,2], Beatriz Hernández-Ochoa [1,3], Gabriel Navarrete-Vázquez [4], Carlos Martínez-Conde [4], Rodrigo Aguayo-Ortiz [5], Fernando Gómez-Chávez [6], Laura Morales-Luna [1,7], Abigail González-Valdez [8], Roberto Arreguin-Espinosa [9], Sergio Enríquez-Flores [10], Verónica Pérez de la Cruz [11], Carlos Wong-Baeza [12], Isabel Baeza-Ramírez [12], and Saúl Gómez-Manzo [2,*]

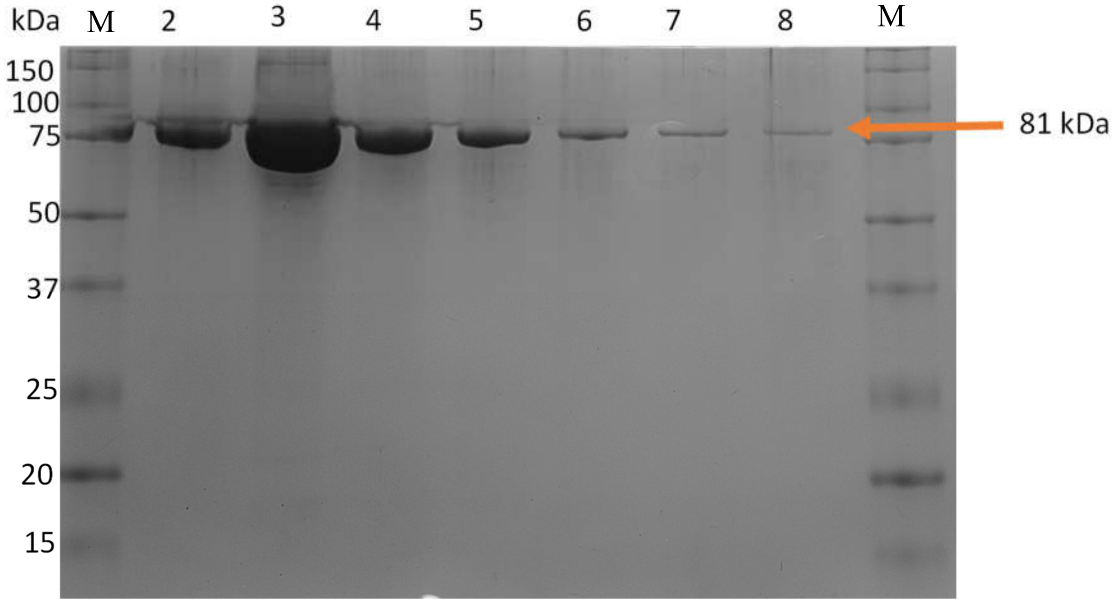

Figure S1. Purification of the recombinant TvG6PD::6PGL enzyme. M: molecular protein weight (MW) marker precision plus protein kaleidoscope standards from Bio-Rad. Lines 1 - 8: 10 µL of protein fractions showed G6PD activity. We stained the gel with a colloidal Coomassie solution. The SDS-PAGE gels are representative of three independent experiments.

Table S1. Data obtained by molecular docking assays with the SwissDock server for the most stable clusters.

Compound	Population	ΔG (Kcal/mol)	Full fitness (Kcal/mol)
**JMM-3 Zone 1**	16	-7.0833	-3770.4844
**JMM-3 Zone 2**	216	-8.6520	-3770.0078
**MCC-7 Zone 1**	64	-8.0406	-3867.0112
**MCC-7 Zone 2**	112	-7.3288	-3866.477

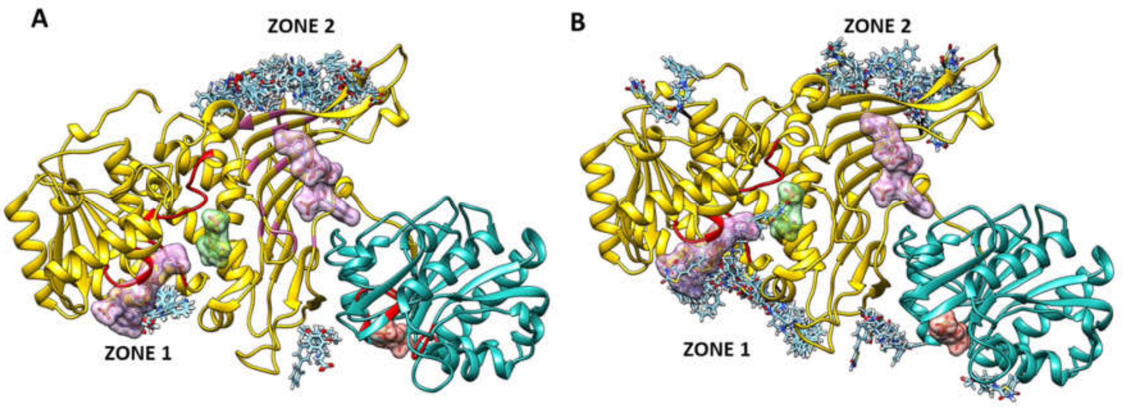

Figure S2. Molecular docking of the TvG6PD::6PGL model with compounds obtained with the SwissDock server. (**A**) General view of the binding affinities of all JMM-3 conformers with the TvG6PD::6PGL protein (**B**) General view of the binding affinities of all MCC-7 conformers with the TvG6PD::6PGL protein. The G6PD and 6PGL are shown in gold and light sea green color.
